# Serum Levels of T Cell Immunoglobulin and Mucin-Domain Containing Molecule 3 in Patients with Systemic Lupus Erythematosus

**DOI:** 10.3390/jcm9113563

**Published:** 2020-11-05

**Authors:** Tomoyuki Asano, Naoki Matsuoka, Yuya Fujita, Haruki Matsumoto, Jumpei Temmoku, Makiko Yashiro-Furuya, Shuzo Sato, Eiji Suzuki, Hiroko Kobayashi, Hiroshi Watanabe, Kiyoshi Migita

**Affiliations:** Department of Rheumatology, Fukushima Medical University School of Medicine, 1 Hikarigaoka, Fukushima, Fukushima 960-1295, Japan; asanovic@fmu.ac.jp (T.A.); naoki-11@fmu.ac.jp (N.M.); fujita31@fmu.ac.jp (Y.F.); haruki91@fmu.ac.jp (H.M.); temmoku@fmu.ac.jp (J.T.); myashiro@fmu.ac.jp (M.Y.-F.); shuzo@fmu.ac.jp (S.S.); azsuzuki@ohta-hp.or.jp (E.S.); hkoba@fmu.ac.jp (H.K.); chiehiro@fmu.ac.jp (H.W.)

**Keywords:** galectin 9, systemic lupus erythematosus, T cell immunoglobulin domain and mucin-domain-containing molecule 3

## Abstract

Objective: T cell immunoglobulin and mucin-domain-containing molecule 3 (TIM-3) is implicated in the development of various autoimmune diseases. We aimed to investigate the levels of soluble TIM-3 (sTIM-3) and their associations between clinical parameters in patients with systemic lupus erythematosus (SLE). Methods: Serum samples were collected from 65 patients with SLE and 35 age-matched healthy controls (HCs). The SLE Disease Activity Index 2000 (SLEDAI-2K) and the Systemic Lupus International Collaborating Clinics (SLICC) damage index (SDI) were used to assess SLE disease activity and SLE-related organ damage. British Isles Lupus Assessment Group (BILAG)-2004 index was also used to assess SLE disease activity. Soluble TIM-3 (sTIM-3) in sera from patients with SLE and HCs were evaluated by enzyme-linked immunosorbent assay (ELISA). The results were compared with the clinical parameters of SLE including SLE disease activity. Results: Serum sTIM-3 levels in patients with SLE (median 2123 pg/mL (interquartile range (IQR), 229–7235)) were significantly higher than those in HCs (1363 pg/mL; IQR, 1097–1673; *p* = 0.0015). Serum levels of sTIM-3 were correlated with disease activity of SLE using the SLEDAI-2K score (*p* < 0.001, *r* = 0.53). The serum sTIM-3 levels in SLE patients with active renal disease (BILAG renal index A-B) were significantly higher than those without the active renal disease (BILAG renal index C–E). However, no significant difference was observed in serum sTIM-3 levels between SLE patients with and without active involvement in other organs (BILAG index). Serum sTIM-3 levels were significantly elevated in SLE patients with organ damage (2710 pg/mL; IQR, 256–7235) compared to those without organ damage (1532 pg/mL; IQR, 228–5274), as assessed by the SDI (*p* = 0.0102). Conclusions: Circulating sTIM-3 levels are elevated in SLE patients, and serum sTIM-3 levels are associated with SLE disease activity and SLE-related organ damage. The data indicate a possible link between the TIM-3/Gal-9 pathway and SLE clinical phenotypes, and further investigation of the TIM-3 pathway in SLE pathophysiology is warranted.

## 1. Introduction

Immune checkpoint receptors of co-inhibitory or co-stimulatory molecules are major components of the immune system [1]. As a negative checkpoint receptor, T cell immunoglobulin and mucin-domain-containing molecule 3 (TIM-3) and its ligand galectin 9 (Gal-9) are thought to be involved with the pathogenesis of autoimmune diseases [2]. TIM-3 is a transmembrane glycoprotein mainly expressed in Th1 and Th17 cells [3]. TIM-3 plays a central role in immune tolerance because the TIM-3/Gal-9 pathway regulates Th1 immunity through apoptosis induction [4], and blocking this interaction results in exacerbated autoimmunity [5]. Systemic lupus erythematosus (SLE) is a systemic autoimmune disease characterized by autoantibody production, immune complex deposition, and cytokine activation [6]. Although the etiology of SLE is complex, it is thought that disrupted self-tolerance leads to activate autoreactive T cells, which subsequently promote auto-antibody production by autoreactive B cells [7]. The role of immune co-inhibitory or co-inhibitory systems in anti-tumor immune responses has been demonstrated to be important by the recent success of immune checkpoint blockade in cancer therapy [8]. However, blocking inhibitory immune checkpoint receptors often causes immune-mediated adverse events (irAEs), which are similar to autoimmune diseases [9]. Conversely, blocking immune responses by enhancing co-inhibitory signals or blocking co-stimulatory signals is a promising therapeutic approach for treating autoimmune diseases [10]. In SLE patients, the TIM-3 ligand, Gal-9, is upregulated and correlates with interferon-signature gene expression [11]. These findings indicate that the TIM-3/Gal-9 pathway plays an important role in Th1 and Th17 immune response and blockage of the TIM-3/Gal-9 interaction results in exacerbated autoimmune diseases [12]. Expression of TIM-3 on peripheral blood mononuclear cells (PBMCs) isolated from SLE patients is associated with SLE disease activity [13]. TIM-3 can be shed from the cell surface by a-disintegrin-like and metalloproteinase with thrombospondin type 1 motifs (ADAM) 10 or ADAM17-mediated cleavage within the TIM-3 stalk region, resulting in a soluble form of TIM-3 (sTIM-3) [14], which is elevated in the sera of patients with autoimmune diseases [15]. In the present study, we quantified circulating sTIM-3 in both patients with SLE and healthy control subjects. We also investigated the associations between circulating sTIM-3 and clinical parameters of SLE, including disease activity.

## 2. Materials and Methods

### 2.1. Patients and Clinical Evaluations

All patients enrolled in this cohort study were diagnosed with SLE at the Department of Rheumatology, Fukushima Medical University Hospital, from June 2009 to September 2019. The enrolled SLE patients had to be older than 17 years to be diagnosed as SLE according to the American College of Rheumatology (ACR) 1997 criteria [16]. A total of 65 Japanese patients with SLE were recruited within 32 months (mean 18 months, range 0–32) from diagnosis with SLE. In patients with SLE, their medical histories and clinical findings were collected by reviewing electronic medical records. Disease activity of SLE was assessed according to the Systemic Lupus Erythematosus Disease Activity Index (SLEDAI) [17]. Chronic organ damage was assessed by the Systemic Lupus International Collaborating Clinics (SLICC) damage index (SDI) [18]. Disease activity of SLE was also assessed using the British Isles Lupus Assessment Group (BILAG)-2004 index, which was proposed to assess the disease activity of SLE using eight systems [19]. The total BILAG-2004 index was calculated by assigning the following numerical values to each BILAG index (BILAG Grade *A* = 12, *B* = 5, *C* = 1, *D* = 0, *E* = 0). Thirty-five healthy controls (HCs) (14 men, 21 women (median age 42 years); interquartile range (IQR), 35–52) were included in this cohort. Ethical approval was obtained for this study from the Committee of Fukushima Medical University International Review Board (No. 30285). Written informed consent was obtained from each individual. All research was performed under the principles of the Declaration of Helsinki.

### 2.2. Enzyme-Linked Immunosorbent Assay for Soluble TIM-3

Serum sTIM-3 levels from patients with SLE and HCs were measured by the human enzyme-linked immunosorbent assay (ELISA) kit (R&D Systems, Inc. Minneapolis, MN, USA), according to the manufacturer’s instructions.

### 2.3. Statistical Analysis

Results were non-normally distributed, and they are presented as median and 25th and 75th percentiles (median (IQR)). A nonparametric test by Mann–Whitney’s U test was used to determine the statistical significance of the data. Spearman’s rank correlation test was used to test the correlations between serial variables.

## 3. Results

### 3.1. Demographic and Clinical Characteristics of SLE Patients

Sixty-five patients with SLE and 35 healthy control subjects (HCs) were included in this study. The overall baseline demographic and clinical characteristics of the SLE patients are summarized in Table 1. Of these, 55 (84%) of SLE patients were women and 10 (16%) were men, median age was 33 years (range 16–79), and median disease duration was 55 months (range 1–420). Sixty (92%) patients had baseline SLEDAI-2K > 4 and 37 (57%) patients had organ damage (SDI ≥ 1). There were no statistically significant differences in sex or age distribution between patients with SLE and HCs.

### 3.2. Correlation between Serum sTIM-3 Levels and Disease Activity of SLE

**Figure 1:**
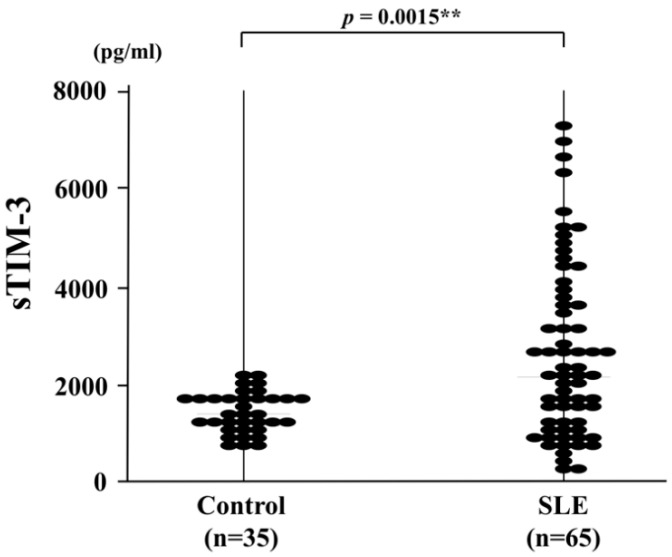
Serum sTIM-3 levels in SLE patients (*n* = 65) and healthy controls (*n* = 35). Soluble TIM-3 levels of SLE patients were significantly higher than those in healthy controls. Median sTIM-3 levels (bar) are displayed and statistical analysis was conducted using the Mann–Whitney’s U test. sTIM-3: soluble T cell immunoglobulin and mucin-domain-containing molecule 3, SLE: systemic lupus erythematosus. **, *p* < 0.01 is considered as statistically significant.

**Figure 2:**
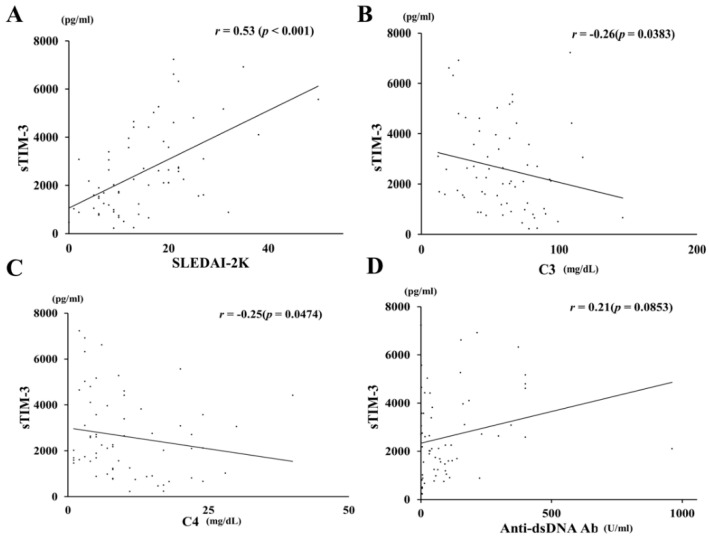
Correlations between serum sTIM-3 levels and clinical parameters in SLE patients. Serum sTIM-3 levels were significantly positively correlated with SLEDAI-2K (**A**) and negatively correlated with serum levels of C3 (**B**) and C4 (**C**). No significant correlation was observed between serum sTIM-3 levels and anti-double-stranded (ds) DNA antibodies (**D**). Statistics and regression lines are shown by solid lines. sTIM-3: soluble T-cell immunoglobulin and mucin-domain-containing molecule 3, SLEDAI-2K: Systemic Lupus Erythematosus Disease Activity Index 2000, C3: complement 3, C4: complement 4.

sTIM-3 levels in sera were determined by ELISA in patients with SLE and HCs. Soluble TIM-3 levels were significantly higher in patients with SLE (2123 pg/mL (IQR, 229–7235)) than those in HCs (1363 pg/mL; IQR, 1097–1673; *p* = 0.0015; Figure 1). We also analyzed the correlations between sTIM-3 levels and SLE disease activity. Serum sTIM-3 levels showed a significant correlation with disease activity when using the SLEDAI-2K (Figure 2A, *p* < 0.001, *r* = 0.53). Serum sTIM-3 levels showed a negative correlation with serum levels of complement (C) 3 (Figure 2B, *p* = 0.038, *r* = −0.26) and complement (C) 4 (Figure 2C, *p* = 0.047, *r* = −0.25), but not with anti-double stranded-DNA antibody titer (Figure 2D, *p* = 0.085, *r* = 0.21).

### 3.3. Serum Levels of sTIM-3 and Organ Involvement Measured by the BILAG-2004 Index

**Figure 3:**
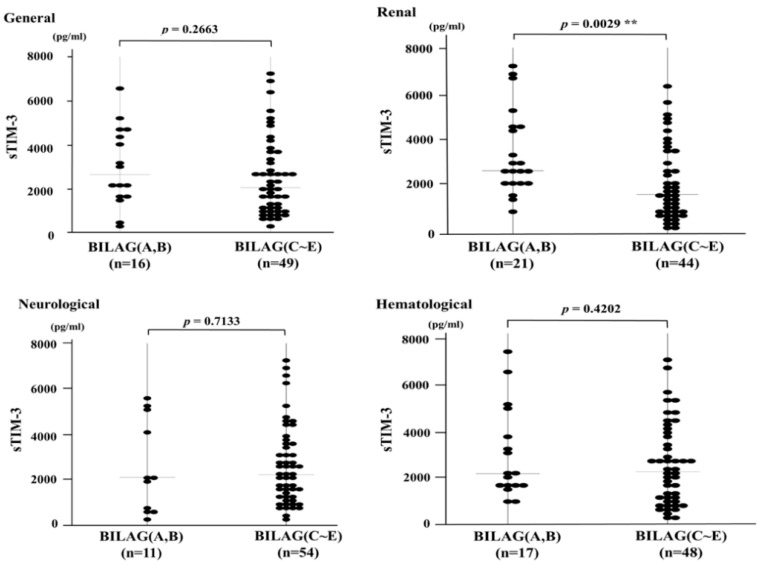
Serum sTIM-3 levels in patients SLE (systemic lupus erythematosus) patients with or without active organ involvement. Comparison of serum sTIM-3 levels between SLE patients with active organ involvement (BILAG general, renal, neurological, and hematological indexes A–B) and without active organ involvements (BILAG index C–E). BILAG: British Isles lupus assessment Group, sTIM-3: soluble T cell immunoglobulin and mucin-domain-containing molecule 3. **, *p* < 0.01 is considered as statistically significant.

The BILAG-2004 index categorizes disease activity into five different levels from A to E, in which A represents the most active form of the disease and E implies the system has never been active [20]. Among the eight BILAG grading domains, active organ involvement was identified mainly in the renal, neurological, and hematological domains (Table 2). The serum sTIM-3 levels in SLE patients with active renal involvement (BILAG index A–B) were significantly elevated compared to those without active renal involvement (BILAG index C–E) (Figure 3). However, no significant difference was observed in serum sTIM-3 levels between SLE patients with or without active neurological or hematological involvement.

We compared the serum levels of sTIM-3 between SLE patients with and without proteinuria. Serum levels of sTIM-3 were significantly higher in SLE patients with proteinuria compared to those without proteinuria (proteinuria negative; *n* = 20; 1361 pg/mL; IQR, 889–2114, versus proteinuria positive; *n* = 35; 2711 pg/mL; IQR, 2108–4536; *p* < 0.001). Similarly, serum levels of sTIM-3 were significantly elevated in SLE patients with microhematuria compared to those without microhematuria (microhematuria negative; *n* = 39, 1693 pg/mL, IQR, 896–2644, versus microhematuria positive; *n* = 26; 2911 pg/mL; IQR, 2156–5139; *p* < 0.001).

### 3.4. Circulating Levels of sTIM-3 and Organ Damage Measured by the SDI

**Figure 4:**
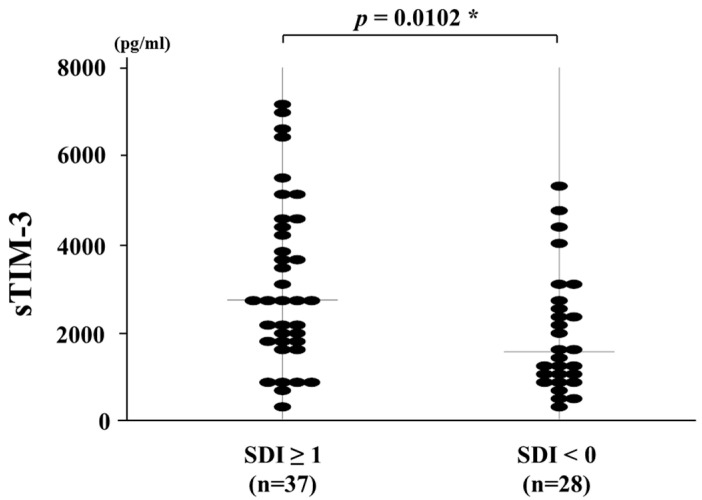
Serum sTIM-3 levels in SLE patients with or without organ damage. Comparison of serum sTIM-3 levels between SLE patients with at least one organ damaged (SDI ≥ 1) and those without organ damage (SDI = 0). Raised serum sTIM-3 levels were shown in SLE patients with at least one organ damage compared with those without organ damage. sTIM-3: soluble T-cell immunoglobulin and mucin-domain-containing molecule 3, SDI: Systemic Lupus International Collaborating Clinics/American College of Rheumatology Damage Index. *, *p* < 0.05 is considered as statistically significant.

Finally, we compared serum sTIM-3 levels between SLE patients with or without organ damage (Figure 4). SLE patients were subdivided into two groups based on the presence of at least one organ damage (SDI ≥ 1) (*n* = 37). In SLE patients with SDI ≥ 1, involvement of the ocular (*n* = 11, 29%), renal (*n* = 8, 21%), neuropsychiatric (*n* = 6, 16%), musculoskeletal (*n* = 5, 13%), and respiratory (*n* = 5, 13%) domains were most prevalent. Serum sTIM-3 levels were significantly elevated in SLE patients having at least one organ damaged (SDI ≥ 1) (2710 pg/mL; IQR, 256–7235) than those without organ damage (SDI = 0) (1532 pg/mL; IQR, 228–5274; *p* = 0.0102).

## 4. Discussion

TIM-3 is expressed on the surface of terminally differentiated T cells and has been implicated in the pathogenesis of Th1-driven autoimmune diseases by negatively regulating the T cell response [21]. The identification of Gal-9 as a ligand for TIM-3 revealed that the TIM-3/Gal-9 pathway is an important regulator of Th1 immunity and immune tolerance [22]. SLE is a Th1-dependent human autoimmune disease that is characterized by autoantibody production and immune complex deposition [23]. Although relationships between SLE and immune co-signaling pathways have been identified, TIM-3/Gal-9 interactions in the pathogenesis of SLE have yet to be clarified.

In the present study, we demonstrated that serum sTIM-3 levels were significantly higher in SLE patients than in HCs. We found a positive correlation between serum sTIM-3 levels and SLE disease activity. Additionally, serum sTIM-3 levels were significantly higher in SLE patients with active renal involvement compared with patients without renal lesions. Thus, the relationship between serum sTIM-3 and SLE disease activity or SLE-related organ involvement indicates that serum sTIM-3 can reflect the SLE disease activity. Higher disease activity in SLE patients can increase the risk of subsequent organ damage [24]. Furthermore, circulating sTIM-3 levels can be used to discriminate between SLE patients with and those without organ damage, as measured by the SDI. Accumulated organ damage, which was measured with the SDI, seems to be related to cumulative disease activity over time or the frequency of severe disease flares. However, this damage index can be influenced by causes that are disease- or treatment-related or the result of concomitant disease. The results indicated that serum levels of sTIM-3 are notable as a clinically useful biomarker and can be a predictor for SLE disease activity.

The TIM-3/Gal-9 coinhibitory pathway is thought to be an important modulator of autoimmunity [5]. In SLE patients, the CD3^+^CD4^+^TIM3^+^ T cell subset was shown to be increased compared with those in HCs, and TIM-3 expression on T cells correlates with SLE disease activity [25]. Elevated serum levels of Gal-9 were also demonstrated in SLE patients [11,26]. It can be concluded that the TIM-3/Gal-9 pathway activation works in SLE patients as an anti-immune mediator. The administration of intraperitoneal Gal-9 to lupus-prone mice ameliorated their proteinuria and arthritis by decreasing anti-double stranded-DNA antibody levels [27]. However, this pathway can be modulated by the sTIM-3 that is shed from TIM-3 expressed on the surface of the immune cells [28]. TIM-3/Gal-9 interaction may result in T cell exhaustion; in contrast, sTIM-3 seems to have alternative effects against this feedback mechanism. Of note, a soluble form of a receptor may not always result in receptor blockage; further studies are needed to determine the source of serum sTIM-3 and the role of sTIM-3 in SLE pathophysiology.

Jin et al. found an increased level of serum sTIM-3 in SLE patients that was correlated with increased serum IL-17 levels [29]. They also reported that serum sTIM-3 levels were significantly lower in SLE patients with lupus nephritis and that there was no significant correlation between sTIM-3 levels and SLEDAI scores [29]. In contrast, our data showed a positive correlation between serum levels of sTIM-3 and SLEDAI scores or active renal involvement determined by BILAG-2004 scores. These discrepancies could be caused by the variations in the demographic data of the SLE patients studied; in our study, we enrolled untreated patients and patients with active SLE. A further longitudinal study with a large number of SLE patients with different disease phenotypes is required to determine the role of sTIM-3 in SLE pathophysiology.

However, three major limitations should be noted in the present study. First, the sample size was relatively small, which limited the statistical power of this study. Second, our study did not address the organ damage of SLE specifically, which would require a repeated assessment of SLE disease activity over a longer period. Third, the mechanism through which TIM-3/Gal-9 pathway contributes to the pathogenesis of SLE was not clarified. Further research involving a large sample size is required to evaluate the usefulness of sTIM-3 determination in patients with SLE.

## 5. Conclusions

Our data indicate that patients with SLE have higher circulating levels of sTIM-3 compared with healthy subjects and that sTIM-3 levels correlate with SLE disease activity. These findings indicate a close association between circulating sTIM-3 and active SLE or particular SLE-related organ involvement. This highlights the need for future research to clarify how this association contributes to the development of SLE.

## Figures and Tables

**Table 1:** General characteristics of 65 Japanese systemic lupus erythematosus patients.

Characteristics	*n* = 65
Sex	
Female, *n* (%)	56 (84)
Male, *n* (%)	11 (16)
Median age (range), years	33 (16–79)
Median disease duration of SLE (range), months	55 (1–420)
Untreated patients, *n* (%)	55 (83)
Items of SLE classification criteria, *n* (%)	
Rash	32 (48)
Alopecia	3 (4)
Oral ulcer	5 (7)
Arthritis	22 (33)
Serositis	17 (25)
Renal disorder	38 (57)
Laboratory findings, *n* (%)	
Leukocytopenia	25 (37)
Thrombocytopenia	18 (27)
Anti-double stranded-DNA antibody positive	52 (78)
Anti-smith antibody positive	31 (46)
Anti-phospholipid antibody positive	28 (42)
Median SLEDAI, index (range)	14 (0–50)
Median SDI, index (range)	1 (0–4)

SLE: systemic lupus erythematosus, SLEDAI: SLE disease activity index, SDI: Systemic Lupus International Collaborating Clinics (SLICC) damage index.

**Table 2:** Disease activity of SLE patients using the British Isles Lupus Assessment Group (BILAG)-2004 index.

BILAG	Grade (*n* = 65)				
Manifestations	A	B	C	D	E
General	3	14	16	4	29
Mucocutaneous	1	5	19	6	35
Neurological	2	10	1	1	52
Musculoskeletal	2	4	14	4	42
Cardiovascular/respiratory	0	6	9	3	48
Abdominal	1	7	0	1	57
Renal	15	6	6	5	34
Hematological	2	15	36	3	10
Ophthalmic	1	4	6	2	53

BILAG grades A: severe, B: intermediate, C: mild, D: inactive, E: no activity.

## Abbreviations

ADAMTS	a disintegrin and metalloproteinase with a thrombospondin type 1 motif
BILAG-2004	British Isles Lupus Assessment Group 2004
IQR	Interquartile range
Gal-9	Galectin-9
SLE	Systemic lupus erythematosus
SLEDAI	Systemic Lupus Erythematosus Disease Activity Index
SLICC	Systemic Lupus International Collaborating Clinics
TIM-3	T cell immunoglobulin and mucin-domain containing 3
